# Understanding community pharmacists’ intentions to report adverse drug reactions in Saudi Arabia: a theory of planned behavior analysis

**DOI:** 10.3389/fphar.2025.1574412

**Published:** 2025-05-20

**Authors:** Fahad T. Alsulami, Yousef Saeed Alqarni, Wadia Saad Alruqayb, Mohammed S. Alharthi, Mohammad S. Alzahrani, Majed A. Algarni, Musaad M. Althobaiti, Saad Mohammed Almalki, Nawaf Abid Altowarqi, Hussain Ali Mathkur, Anas Muslih Althumali, Ayman Mohammed Alharthi, Haifa Abdulrahman Fadil, Adel A. Altowairqi, Mubarak S. Alharthi

**Affiliations:** ^1^ Clinical Pharmacy Department, College of Pharmacy, Taif University, Taif, Saudi Arabia; ^2^ Department of Pharmacy Practice, College of Pharmacy, Imam Abdulrahman Bin Faisal University, Dammam, Saudi Arabia; ^3^ Department of Pharmacology and Toxicology, College of Pharmacy, Taif University, Taif, Saudi Arabia; ^4^ College of Pharmacy, Taif University, Taif, Saudi Arabia; ^5^ Department of Pharmacy practice, Faculty of Pharmacy, Taibah University, Almadinah Almunawarah, Saudi Arabia; ^6^ University Medical Clinics, Taif University, Taif, Saudi Arabia

**Keywords:** adverse drug reaction, community pharmacist, pharmacovigilance, theory of planned behavior, Saudi Arabia

## Abstract

**Introduction:**

Adverse drug reaction (ADR) reporting is critical for ensuring medication safety. However, underreporting remains a global concern, particularly in community pharmacy settings. This study explores the behavioral factors influencing community pharmacists’ intention to report ADRs in Saudi Arabia, using the Theory of Planned Behavior (TPB) as a guiding framework.

**Aims and Objectives:**

This study aimed to estimate factors affecting community pharmacists’ intention to report ADRs to the Saudi National Pharmacovigilance Center (NPC) through a TPB-based analysis.

**Methods:**

A cross-sectional survey was conducted to collect data on pharmacists’ intentions, attitudes, subjective norms, perceived behavioral control, and moral obligation regarding ADR reporting. Descriptive statistics summarized the sample characteristics. Binary logistic regression was used to evaluate the influence of TPB constructs on reporting intentions.

**Results:**

Among 452 participating community pharmacists, 88% were aware of the NPC, but only 27.4% had reported ADRs in the past year. Pharmacists who were non-Saudi, aware of the NPC and its procedures, and trained in ADR reporting demonstrated significantly higher intentions to report (p < 0.05). Attitudes (OR = 1.141, 95% CI: 1.09–1.18, p < 0.001), subjective norms (OR = 1.280, 95% CI: 1.16–1.40, p < 0.001), perceived behavioral control (OR = 1.168, 95% CI: 1.03–1.31, p = 0.010), and moral obligation (OR = 1.417, 95% CI: 1.05–1.89, p = 0.019) were all significantly associated with reporting intention.

**Conclusion:**

Findings reinforce the importance of targeting TPB constructs (particularly attitudes, social norms, and perceived control) and perceived moral obligation in designing interventions to improve ADR reporting. Strategies such as structured training, institutional support, and promoting moral responsibility may bridge the gap between awareness and actual reporting practices among community pharmacists in Saudi Arabia.

## 1 Introduction

Adverse drug reactions (ADRs) continue to be major causes of morbidity and mortality (Lazarou et al., 1998; Dc et al., 1997). Compared to other significant factors contributing to mortality, such as heart disease, cancer, and stroke, ADRs are approximated to be among the fourth to sixth leading causes of death worldwide (Bates, 1998; Bonn, 1998). In England, the number of hospitalizations caused by ADRs increased from 1.2% in 2008 to 1.6% in 2015 (Veeren and Weiss, 2017). In Saudi Arabia, there were 8.5 per 100 admissions related to ADRs in one hospital (Aljadhey et al., 2013). Additionally, ADRs place a significant burden on the economy (Dormann et al., 2000). It is predicted that the expense of managing ADRs in the United States will be as high as 30.1 billion dollars (Sultana et al., 2013). In Europe, the costs for handling ADRs are thought to be €79 billion (Leporini et al., 2014). The World Health Organization (WHO) characterizes ADRs as any negative consequence that is unforeseen and not expected, resulting from the use of a medication as prescribed (Coleman and Pontefract, 2016).

Timely and precise reporting of ADRs plays a crucial role in overseeing the safety and effectiveness of pharmaceutical products. It empowers healthcare authorities to implement necessary measures, minimizing risks and promoting patient safety (Brewer and Colditz, 1999). According to WHO, pharmacovigilance involves various activities related to the detection, assessment, and avoidance of ADRs (Zdrowia, 2002). However, one of the biggest challenges facing pharmacovigilance programs is inadequate reporting (Alharf et al., 2018). The global reporting rate for ADRs is estimated to be between 5% and 10%. Thus, under-reporting is a real concern that we must consider (Sultana et al., 2013).

The Saudi Food and Drug Authority (SFDA) established the National Pharmacovigilance Center (NPC) in 2009 in Saudi Arabia (Alshammari et al., 2017). The NPC has established both paper and online methods to make ADR reporting more accessible and expects all healthcare practitioners, including doctors, pharmacists, and nurses, to do so. However, ADRs are still to be underreported by healthcare providers (HCPs) in Saudi Arabia (Alshammari et al., 2017). Saudi Arabia, with other Middle Eastern countries, accounts for only 0.6% of the world’s safety reporting (Ahmad, 2014).

Pharmacists are the most qualified HCPs to identify and report ADRs as they are the experts in drugs and the most accessible healthcare practitioners (Van Grootheest and De Jong-van den Berg, 2005). Over time, a pharmacist’s responsibilities within the healthcare system have expanded beyond the traditional dispensing duties to increasing engagement in patient therapy through the provision of pharmaceutical care (Hepler and Strand, 1990). However, pharmacists made less contributions to reporting ADRs, and they were more hesitant to report the ADRs compared with physicians (Lemay et al., 2018). In Saudi Arabia, while most pharmacists observed 6 to 10 ADRs during their practice in hospital and community settings, most were unwilling to report ADRs to NPC (Khan, 2013; Alshabi et al., 2022). Several barriers to reporting ADRs have been identified by pharmacists in Saudi Arabia, including lack of awareness about NPC, lack of knowledge about who to report ADRs, and lack of incentives, access, and time to report ADRs (Alshabi et al., 2022; Mahmoud et al., 2014; Al Doughan et al., 2019; Alsheikh and Alasmari, 2022). Community pharmacists serve as highly accessible healthcare professionals who maintain active engagement within their communities, enabling their services to reach a broad segment of the population (Alfadl et al., 2018). As primary care providers, they play a crucial role in delivering healthcare guidance and are often the first point of contact for individuals seeking medical advice. Given their accessibility, community pharmacists represent a valuable source of information on ADRs. When consumers report suspected ADRs, pharmacists can conduct an initial assessment, screening the reported cases before submitting them to the pharmacovigilance reporting systems, thereby enhancing the accuracy and efficiency of ADR monitoring (Hughes and Weiss, 2019; Li et al., 2018).

While few studies evaluate the effect of awareness and knowledge about NPC in reporting ADRs among community pharmacists in Saudi Arabia, no known research applied a theoretical framework to guide the empirical investigation of community pharmacists’ ADRs reporting behavior in Saudi Arabia. Furthermore, little is known about the primary influencers of the community pharmacists’ behavior and intention to report ADRs and the actual contributions made by these causes in predicting the intention and behavior. This research fills a knowledge gap in the existing literature by employing the theory of planned behavior (TPB) as a conceptual framework to enhance our comprehension of the reporting behavior of pharmacists regarding ADRs.

The TPB is a cognitive theory that predicts cognitive factors associated with intention and behavior. It aims to understand and forecast human actions by investigating how attitudes, subjective norms, and perceived behavioral control shape behavior. Attitude refers to the extent to which an individual views the behavior of interest positively or negatively. Subjective norms involve the perceived social pressure and influence exerted by important individuals to either participate in or abstain from a particular behavior. Lastly, perceived behavioral control involves an individual’s evaluation of their capacity to carry out a particular behavior, taking into account elements like their skills, available resources, and external obstacles. These three components collectively shape an individual’s intention, which in turn guides their actual behavior. TPB has proven to be a valuable tool in various fields, including health psychology, marketing, and social sciences, providing insights into how interventions can be designed to influence and modify human behavior effectively (Ajzen, 1991). Beyond the three core constructs of the TPB (attitude, subjective norms, and perceived behavioral control) perceived moral obligation has been identified as a significant determinant of behavioral intention. Empirical evidence suggests that the explanatory power of the TPB framework can be enhanced by incorporating additional variables, such as moral norms or perceived moral obligation (Werner and Mendelsson, 2001; Godin et al., 2008). A comprehensive meta-analysis has further substantiated the value of integrating perceived moral obligation into the TPB, demonstrating its relevance across various health-related behaviors (Conner and Armitage, 1998). In the context of ADR reporting, several researchers have advocated for the inclusion of perceived moral obligation as a predictor of pharmacists’ intentions to report (Gavaza et al., 2011; Raghvan and Fatokun, 2021). Consistent with this perspective, the present study extends the TPB framework by incorporating perceived moral obligation, hypothesizing that it contributes meaningfully to the prediction of community pharmacists’ reporting intentions regarding ADRs.

This study aimed to investigate the factors influencing community pharmacists’ intentions to ADRs in Saudi Arabia. Specifically, it examined the predictive role of the TPB constructs and perceived moral obligation on reporting intentions. Additionally, the study assessed community pharmacists’ awareness of the NPC, the ADR reporting system, and their actual ADR reporting practices.

## 2 Methods

### 2.1 Sample size and sampling methods

The sample size was determined using the Raosoft sample size calculator (http://www.raosoft.com/samplesize.html). Given that the total number of community pharmacists in Saudi Arabia was 10,347, the target sample comprised at least 371 community pharmacists, calculated using a 95% confidence level and a 5% margin of error (Alrasheedy, 2023).

A cross-sectional survey was conducted online using a convenience sampling method to target community pharmacists across Saudi Arabia. The self-administered questionnaire was distributed through Google Forms, with recruitment efforts facilitated *via* social media platforms such as WhatsApp and Telegram. Additionally, pharmacy students were enlisted to share the survey link with eligible participants, and chain community pharmacies were approached to help disseminate the survey to their pharmacist employees. This approach was chosen due to its feasibility in reaching a geographically dispersed population, as well as time and resource constraints, and the lack of a centralized database of community pharmacists in Saudi Arabia. While convenience sampling has inherent limitations, it enabled efficient data collection from a diverse sample of community pharmacists across various locations.

The inclusion criteria required participants to be registered pharmacists in Saudi Arabia and currently employed in a community pharmacy setting. The exclusion criteria included pharmacists not actively practicing in a community pharmacy, such as those working in hospitals, academia, or administrative roles, as well as those not licensed to practice in Saudi Arabia.

### 2.2 Study instruments

A structured questionnaire, adapted from previously validated tools, was used in this study (Gavaza et al., 2011; Raghvan and Fatokun, 2021). It included sections on sociodemographic data, awareness of the NPC and ADR reporting systems, community pharmacists’ ADR reporting practices, and constructs from the TPB. A section on perceived moral obligation was also included.

The survey was developed based on a literature review and refined following feedback from two academic experts. A pilot study involving 10 community pharmacists (whose data were excluded from the final analysis) was conducted to assess the questionnaire’s face and content validity. To enhance clarity and avoid linguistic misunderstandings, the survey was made available in both English and Arabic.

Cronbach’s alpha was used to assess the internal consistency of the TPB constructs. The results indicated high reliability, with Cronbach’s alpha values ranging from 0.846 to 0.923. Based on feedback from the pilot study, minor modifications were made to improve clarity, but no substantial changes were necessary.

#### 2.2.1 TPB constructs

The TPB framework was operationalized through the measurement of four key constructs: intention, attitude, subjective norms, and perceived behavioral control. All items used to assess these constructs were adapted from previously validated scales (Gavaza et al., 2011; Raghvan and Fatokun, 2021). Community pharmacists’ intention to report ADRs was measured using three items (e.g., “I intend to report serious ADRs that I encounter to the Saudi NPC”). Responses were captured using a seven-point Likert scale ranging from “strongly disagree” to “strongly agree.” The total score for the intention construct ranged from three to 21. A higher score indicated a stronger intention to report ADRs to the NPC. For analysis purposes, the mean score was used as a cut-off point to dichotomize the intention variable: pharmacists scoring below the mean were coded as having low intention (0), while those scoring at or above the mean were coded as having high intention (1). This binary variable served as the dependent variable in logistic regression models.

Attitudes toward reporting ADRs were assessed using five items (e.g., “Reporting ADRs to the NPC in Saudi Arabia is valuable”). Responses were captured using the same seven-point Likert scale. The total attitude score ranged from −15 to 15, with higher scores indicating a more positive attitude toward ADR reporting. The attitude construct was used as a continuous independent variable in the binary logistic regression.

Subjective norms were measured using three items (e.g., “Most people who are important to me think that I should report ADRs that I encounter to the NPC in Saudi Arabia”). Responses were again captured on a seven-point Likert scale. The total score ranged from −9 to 9, with higher scores indicating that the pharmacist perceived stronger social pressure to report ADRs. This construct was also used as a continuous independent variable in the logistic regression.

Perceived behavioral control was assessed using two items (e.g., “you believe you have complete control over reporting ADRs that I encounter to the NPC in Saudi Arabia”). Responses were recorded using the same seven-point Likert scale, with the total score ranging from −6 to 6. A higher score reflected a greater perceived ability to report ADRs. This construct was likewise used as a continuous independent variable in the binary logistic regression.

#### 2.2.2 Perceived moral obligation

Perceived moral obligation was assessed with a single item (e.g., “I feel morally obligated to report ADRs to the NPC”), using a seven-point Likert scale. This item was adapted from the literature (Gavaza et al., 2011; Raghvan and Fatokun, 2021). The use of a single-item measure is consistent with prior TPB-based studies, where moral obligation is considered a supplementary construct. Although single-item measures may have limitations in terms of internal consistency, they reduce participant burden and are justifiable when the construct is conceptually straightforward. The score ranged from 1 to 7, with higher scores indicating a greater sense of moral responsibility. This variable was treated as a continuous independent variable in the binary logistic regression.

### 2.3 Statistical analysis

Descriptive statistics, including frequencies, percentages, means, and standard deviations, were used to summarize the data. Chi-square tests were used to examine associations between sociodemographic factors and community pharmacists’ intentions to report ADRs. Additionally, binary logistic regression was conducted to assess the influence of TPB constructs and perceived moral obligation on the intention to report ADRs, while controlling for sociodemographic variables. A p-value of less than 0.05 and a 95% confidence interval (CI) were considered statistically significant. All analyses were performed using IBM^®^ SPSS Statistics, version 29.0.

## 3 Results

### 3.1 Respondent profile

A total of 452 community pharmacists completed the online survey. Table 1 summarizes the sociodemographic characteristics of the participants. Approximately 74% were male, with a mean age of 31.2 years. Around 54% of the participants were non-Saudi. The majority of community pharmacists were working in the western region of Saudi Arabia (44.9%), followed by the southern region (21%), central region (18.4%), eastern region (8.4%), and northern region (7.3%). About 70% of participants held a bachelor’s degree in pharmacy, while approximately 30% had a Doctor of Pharmacy (PharmD) degree. More than half (55.1%) reported a monthly income between 7,000 and 10,999 Saudi Riyal. Additionally, more than half of the community pharmacists had less than 5 years of experience.

**TABLE 1:** Sociodemographic characteristics of community pharmacist participants (n = 452).

Sociodemographic variables	Frequency	Percent
Age	Mean: 31.23	SD: 5.66
Gender		
Female	117	25.9%
Male	335	74.1%
Nationality		
Non-Saudi	245	54.2%
Saudi	207	46.8%
Region of work in Saudi Arabia		
Western region	203	44.9%
Eastern region	38	8.4%
Central region	83	18.4%
Northern region	33	7.3%
Southern region	95	21%
Educational Degree		
Bachelor’s degree in pharmacy	315	69.7%
Doctor of Pharmacy (PharmD)	137	30.3%
Monthly Income		
6,999 SAR or less	153	33.8%
7,000 SAR to 10,999 SAR	249	55.1%
11,000 SAR or More	50	11.1%
Number of years practicing in community setting in Saudi Arabia		
Less than 5 years	243	53.8%
From 5 years to 10 years	131	29.0%
More than 10 years	78	17.3%
Are you aware of the existence of NPC in Saudi Arabia?		
Not aware	56	12.4%
Aware	396	87.6%
Are you familiar with the way of ADRs reporting in Saudi Arabia?		
Not familiar	106	23.5%
Familiar	346	76.5%
Have you seen the ADR reporting form before?		
No	182	40.3%
Yes	270	59.7%
Have you been trained to report ADR before?		
No	189	41.8%
Yes	263	58.2%
Have you reported any ADRs to Saudi NPC in the last 12 months?		
No	328	72.6%
Yes	124	27.4%

Approximately 88% of participants were aware of the existence of the NPC in Saudi Arabia, and about 77% were familiar with the process of reporting ADRs to the Saudi NPC. Furthermore, 58% had received training on how to report ADRs, and 60% had seen the ADR reporting form before. However, only 27.4% had reported ADRs to the Saudi NPC in the past 12 months.

### 3.2 Intention to report ADRs

A significant majority of community pharmacists (88%) agreed that they intend to report serious ADRs. Similarly, 88.5% indicated that they would try to report such events. When asked whether they plan to report serious ADRs, 86.7% responded affirmatively. These findings suggest that community pharmacists have a strong intention to report serious ADRs they encounter to the Saudi National Pharmacovigilance Center (NPC), with an overall mean score of 18.68 out of 21. Consequently, approximately 67% of community pharmacists demonstrated a high intention to report ADRs to the Saudi NPC (Table 2).

**TABLE 2:** Intention to report ADRs, TPB constructs, and perceived moral obligation construct among community pharmacist to report ADR to NPC.

Variables	Disagree	Neutral	Agree
Intention *Mean:* 18.6; ranges from 3 to 21; *SD:* 3.8			
1. I intend to report serious ADRs that I will encounter to the Saudi NPC.	5.5%	6.4%	88%
2. I will try to report serious ADRs that I will encounter to the Saudi NPC.	5.8%	5.8%	88.5%
3. I plan to report serious ADRs that I will encounter to the Saudi NPC.	6%	7.3%	86.7%
Attitude *Mean:* 10; ranges from −15 to 15; *SD:* 7			
1. Reporting ADRs to NPC in Saudi Arabia is valuable	11.9%	3.6%	84.5%
2. Reporting ADRs to NPC in Saudi Arabia is pleasant	14.4%	7.3%	78.3%
3. Reporting ADRs to NPC in Saudi Arabia is good	5.9%	7.4%	86.7%
4. Reporting ADRs to NPC in Saudi Arabia is enjoyable	15.9%	10.4%	73.7%
5. Reporting ADRs to NPC in Saudi Arabia is beneficial	9.9%	3.1%	87%
Subjective Norms *Mean:* 5.3 ranges from −9 to 9; *SD:* 4.3			
1. Most people who are important to me think that I should report ADRs that I encounter to NPC in Saudi Arabia	11.7%	9.8%	78.5%
2. The people in my life whose opinions I value would approve my reporting of ADRs that I encounter to NPC in Saudi Arabia	8.2%	9.5%	82.5%
3. The pharmacists whose opinions I value report ADRs to NPC in Saudi Arabia	12.9%	13.9%	73.3%
Perceived Behavioural Control *Mean: 2*.3 ranges from −6 to 6; *SD:* 2.9			
1. You believe you have complete control over reporting ADRs that you encounter to NPC in Saudi Arabia	15%	15%	70%
2. It is mostly up to me whether or not I report ADRs to NPC in Saudi Arabia	21.5%	19.7%	58.8%
Perceived Moral Obligation *Mean:* 6.2 ranges from 1 to 7; *SD:* 1.4			
1. I believe I have a moral obligation to report ADRs that I will encounter to NPC in Saudi Arabia	6.9%	5.5%	87.6%

Note: very strongly disagree to disagree were grouped to disagree category and very strongly agree to agree were grouped to agree category.

Chi-square tests were conducted to identify any significant differences in the rates of community pharmacists with a high intention to report ADRs to the Saudi NPC across different genders, nationalities, regions of work, educational degrees, monthly incomes, levels of awareness regarding the existence of the NPC, familiarity with reporting ADRs, and training in ADR reporting.

As shown in Table 3, approximately 72% of non-Saudi community pharmacists had a high intention to report ADRs to the NPC, compared to about 60% of Saudi pharmacists (p = 0.008). Furthermore, pharmacists with more than 10 years of experience in the community setting had higher rates of intention to report ADRs (82.1%) compared to those with less than 10 years of experience (p = 0.005). Community pharmacists who were aware of the NPC’s existence in Saudi Arabia exhibited higher intention rates (68.9%) than those who were unaware (51.8%) (p = 0.011). Similarly, those familiar with the ADR reporting process showed higher intention rates (70.5%) compared to those unfamiliar with the process (54.7%) (p = 0.003). Moreover, pharmacists who had received training in ADR reporting demonstrated higher intention rates (74.5%) than those who had not been trained (56.1%) (p < 0.001). However, no significant differences were found in the intention to report ADRs among community pharmacists based on gender, region of work, educational degree, or monthly income (p > 0.05).

**TABLE 3:** Community pharmacists with less and high intention to report ADRs to NPC across different sociodemographic variables.

Sociodemographic variables	Community pharmacists with low intention to report ADRs to NPC (n = 150)	Community pharmacists with high intention to report ADRs to NPC (n = 302)	p-value
Gender			0.341
Female	36.8%	63.2%	
Male	31.9%	68.1%	
Nationality			**0.008**
Saudi	39.6%	60.4%	
Non-Saudi	27.8%	72.2%	
Region of work in Saudi Arabia			0.604
Western region	34.5%	65.5%	
Eastern region	39.5%%	60.5%	
Central region	34.9%	65.1%	
Northern region	24.2%	75.8%	
Southern region	29.5%	70.5%	
Educational degree			0.324
Bachelor’s degree in pharmacy	31.7%	68.3%	
Doctor of Pharmacy (PharmD)	35.5%	63.5%	
Monthly income			0.614
6,999 SAR or less	30.7%	69.3%	
7,000 SAR to 10,999 SAR	33.7%	66.3%	
11,000 SAR to 14,999 SAR	38%	62.0%	
Number of years practicing in community setting in Saudi Arabia			**0.005**
Less than 5 years	37.9%	62.1%	
From 5 years to 10 years	33.6%	66.4%	
More than 10 years	17.9%	82.1%	
Awareness of the existence of NPC in Saudi Arabia			**0.011**
Not aware	48.2%	51.8%	
Aware	31.1%	68.9%	
Are you familiar with the way of ADRs reporting in Saudi Arabia?			**0.003**
Not familiar	45.3%	54.7%	
Familiar	29.5%	70.5%	
Have you seen the ADR reporting form before?			**0.018**
No	39.6%	60.4%	
Yes	28.9%	71.1%	
Have you been trained to report ADR before?			**< 0.001**
No	43.9%	56.1%	
Yes	25.5%	74.5%	
Have you reported any ADRs to NPC in the previous 12 months?			0.973
No	33.2%	66.8%	
Yes	33.1%	66.9%	

P-values < 0.05 are highlighted in bold.

### 3.3 TPB constructs and perceived moral obligation

This study included four main predictor variables, including the TPB constructs (attitude, subjective norms, and perceived behavioral control), as well as perceived moral obligation.

The attitude construct was measured using five items. Over 80% of community pharmacists agreed, strongly agreed, or very strongly agreed that reporting ADRs to the NPC is valuable, good, and beneficial. Moreover, more than 70% of them perceived reporting ADRs to NPC as pleasant and enjoyable. The overall mean score for the attitude construct was 10, with a range from −15 to 15, indicating a generally positive attitude toward reporting ADRs to the NPC (Table 2).

The subjective norms construct was measured using three items. Approximately 79% of community pharmacists agreed, strongly agreed, or very strongly agreed that most people important to them believed they should report ADRs they encounter to the NPC. Additionally, about 83% felt that the people whose opinions they valued would approve of them reporting ADRs. Lastly, around 73% agreed that pharmacists whose opinions they valued also reported ADRs to the Saudi NPC. The overall mean score for the subjective norms construct was 5.36, with a range from −9 to 9, indicating that community pharmacists perceived positive subjective norms regarding ADR reporting (Table 2).

The perceived behavioral control construct was measured using two items. Approximately 70% of community pharmacists agreed, strongly agreed, or very strongly agreed that they have complete control over reporting ADRs they encounter to the NPC in Saudi Arabia. Additionally, about 59% of community pharmacists agreed, strongly agreed, or very strongly agreed that it is primarily up to them whether or not they report ADRs to the NPC in Saudi Arabia. The overall mean score for the perceived behavioral control construct was 2.31, with a range of −6 to 6, indicating that community pharmacists perceived a greater degree of behavioral control over reporting ADRs to the NPC (Table 2).

Perceived moral obligation was measured using a single item. Approximately 88% of community pharmacists agreed, strongly agreed, or very strongly agreed that they have a moral obligation to report ADRs encountered to the Saudi NPC. The overall mean score for perceived moral obligation was 6.22, with a range of 1–7. This indicates that community pharmacists exhibited a high level of perceived moral obligation to report ADRs to the NPC (Table 2).

### 3.4 Predictor associated the intention to report ADRs

Binary logistic regression was conducted to identify the effect of TPB constructs (attitude, subjective norms, and perceived behavioral control) and perceived moral obligation on the intention of community pharmacists to report ADRs to the NPC. The model was significant (χ^2^ = 239.78, p < 0.001) and explained approximately 57% of the variance in the pharmacists’ intention to report ADRs (Nagelkerke *R* ^2^ = 0.572). As shown in Table 4, among the demographic factors, neither age (OR = 0.941, 95% CI [0.85–1.03], p = 0.211) nor gender (male vs female, OR = 1.177, 95% CI [0.53–2.57], p = 0.684) had a significant impact. Likewise, nationality (Saudi vs non-Saudi, OR = 1.007, 95% CI [0.33–3.05], p = 0.990) and region of work did not demonstrate a significant effect on reporting intentions. Importantly, income showed some influence, with those earning between 7,000 and 10,999 SAR being less likely to report ADRs compared to those earning less than 6,999 SAR (OR = 0.455, 95% CI [0.23–0.88], p = 0.020). Regarding practice-related factors, years of experience, awareness of the NPC, familiarity with ADR reporting, and prior training in ADR reporting were not significantly associated with reporting intentions.

**TABLE 4:** Predictors associated with community pharmacists’ intention to report ADRs to NPC.

Predictor variables	Odds ratio	Lower 95% CI	Upper 95% CI	p-value
Age	0.941	0.855	1.035	0.211
Gender				
Female	References			
Male	1.177	0.537	2.579	0.684
Nationality				
Non-Saudi	References			
Saudi	1.007	0.332	3.051	0.990
Region of work in Saudi Arabia				
Western region	References			
Eastern region	0.613	0.227	1.650	0.333
Central region	0.946	0.447	2.002	0.885
Northern region	1.596	0.433	5.886	0.483
Southern region	1.398	0.640	3.057	0.401
Educational Degree				
Bachelor’s degree in pharmacy	References			
Doctor of Pharmacy (PharmD)	1.125	0.530	2.388	0.759
Monthly Income				
6,999 SAR or less	References			
7,000 SAR to 10,999 SAR	0.455	0.234	0.884	**0.020**
11,000 SAR or More	0.564	0.207	1.538	0.263
Number of years practicing in community setting in Saudi Arabia				
Less than 5 years	References			
From 5 years to 10 years	1.768	0.636	4.920	0.275
More than 10 years	2.166	0.423	11.102	0.354
Are you aware of the existence of NPC in Saudi Arabia?				
Not aware	References			
Aware	0.883	0.361	2.159	0.785
Are you familiar with the way of ADRs reporting in Saudi Arabia?				
Not familiar	References			
Familiar	0.748	0.317	1.766	0.508
Have you seen the ADR reporting form before?				
No	References			
Yes	0.785	0.375	1.644	0.520
Have you been trained to report ADR before?				
No	References			
Yes	1.878	0.883	3.992	0.102
*TPB Constructs*				
Attitude	1.141	1.096	1.188	**< 0.001**
Subjective Norms	1.280	1.168	1.403	**< 0.001**
Perceived Behavioral Control	1.168	1.038	1.316	**0.010**
*Perceived Moral Obligation*	1.417	1.059	1.895	**0.019**

P-values < 0.05 are highlighted in bold.

After controlling for other variables, the TPB constructs (attitudes, subjective norms, and perceived behavioral control) as well as perceived moral obligation, were significantly associated with the intention of community pharmacists to report ADRs to the NPC. Community pharmacists who held positive attitudes toward reporting ADRs were more likely to have a strong intention to report them (OR = 1.141, 95% CI [1.09–1.18], p < 0.001). Similarly, those who perceived supportive subjective norms were more likely to intend to report ADRs (OR = 1.280, 95% CI [1.16–1.40], p < 0.001). Community pharmacists who felt greater behavioral control regarding ADR reporting also showed a higher intention to report (OR = 1.168, 95% CI [1.03–1.31], p = 0.010). Finally, those who perceived a moral obligation to report ADRs were more likely to have a strong reporting intention (OR = 1.417, 95% CI [1.05–1.89], p = 0.019).

## 4 Discussion

ADRs remain significant contributors to illness and death, ranking among the leading causes of mortality worldwide (Bates, 1998; Bonn, 1998). Accurate and timely reporting of ADRs is essential for monitoring pharmaceutical safety and efficacy, enabling healthcare authorities to implement interventions that enhance patient safety. However, global ADR reporting rates are estimated to be only between 5% and 10%, indicating a concerning level of under-reporting (Sultana et al., 2013). Pharmacists, as medication experts and readily accessible healthcare providers, are well-positioned to identify and report ADRs, thereby playing a critical role in reducing the risks associated with drug therapy (Van Grootheest and De Jong-van den Berg, 2005). This study aimed to assess the rate at which community pharmacists in Saudi Arabia report ADRs and their intention to do so. It employed the TPB to identify cognitive factors influencing their reporting intentions. Additionally, the study examined how awareness of the NPC, familiarity with the reporting process, training in ADR reporting, and perceptions of moral obligation affect pharmacists’ intentions to report ADRs to the NPC.

This study revealed suboptimal rates of ADR reporting among community pharmacists. Approximately 27% of community pharmacists reported ADRs to the NPC in the past 12 months. This finding is consistent with other studies conducted in Saudi Arabia (Al Doughan et al., 2019; Ali et al., 2018). Similarly, low reporting rates have been observed among community pharmacists in other countries (Paut Kusturica et al., 2022; Tomas Petrović et al., 2022). Several barriers to ADR reporting have been identified, including lack of knowledge, high workload, and limited time (Hughes and Weiss, 2019; Paut Kusturica et al., 2022; Al-Mutairi et al., 2021). The low ADR reporting rate observed in this study has significant implications for pharmacovigilance efforts in Saudi Arabia. Underreporting of ADRs limits the ability to detect and prevent medication-related harm, potentially compromising patient safety. Without comprehensive ADR reporting, regulatory agencies may overlook important safety signals, leading to delayed interventions or drug withdrawals. Moreover, the lack of ADR data obstructs post-marketing surveillance and the assessment of medication safety profiles. This study also revealed that over half of community pharmacists (67%) had a strong intention to report ADRs, with the majority (87.6%) being aware of the NPC’s existence in Saudi Arabia. These findings are consistent with other studies conducted in the country (Alsheikh and Alasmari, 2022). They suggest a promising trend in pharmacovigilance within the community pharmacy setting. The high intention to report ADRs indicates a strong commitment among community pharmacists to contribute to drug safety surveillance and improve patient care. Furthermore, the widespread awareness of the NPC reflects the effectiveness of awareness campaigns and educational initiatives aimed at enhancing pharmacists’ understanding of their role in ADR reporting in Saudi Arabia.

This study revealed that community pharmacists who were aware of the existence of the NPC and familiar with the process of reporting ADRs in Saudi Arabia had a significantly greater intention to report ADRs to the NPC than those who were unaware of the NPC or unfamiliar with the reporting process (p < 0.05). Mahmoud and colleagues (2014) noted that a lack of awareness about the ADR reporting process in Saudi Arabia was the most common barrier to reporting among community pharmacists (Mahmoud et al., 2014). These findings underscore the critical role of awareness and familiarity in promoting pharmacovigilance activities among community pharmacists. When pharmacists are knowledgeable about the NPC and understand the ADR reporting procedures in Saudi Arabia, they are more likely to engage in reporting these incidents. This suggests that targeted educational initiatives and awareness campaigns aimed at increasing pharmacists’ understanding of the NPC and ADR reporting protocols could significantly enhance pharmacovigilance efforts in the country. Additionally, it highlights the importance of continuous education and training to ensure pharmacists’ active participation in monitoring and reporting ADRs, ultimately contributing to improved medication safety and patient care.

Furthermore, the study found that community pharmacists who had previously seen the ADR reporting form and received training in ADR reporting were more likely to report ADRs to the NPC compared to those who had neither seen the form nor received training (p-value <0.05). This indicates a significant association between prior exposure to the ADR reporting form and training, and the intention to report ADRs to the NPC. The study’s findings underscore the importance of familiarity with reporting procedures and proper training in enhancing pharmacists’ willingness to report ADRs. These findings suggest that initiatives aimed at educating and familiarizing pharmacists with reporting mechanisms can improve pharmacovigilance efforts and contribute to better patient safety outcomes. Additionally, they highlight the need for ongoing education and support for community pharmacists to ensure sustained and effective ADR reporting practices within the community pharmacy setting.

The study found a lower intention to report ADRs among community pharmacists earning between 7,000 and 10,999 SAR. This finding could be attributed to several possible factors. First, economic pressures may play a role. Community pharmacists in this income range might have financial obligations that require them to work more efficiently, potentially reducing the time and motivation available for optional tasks such as ADR reporting. Second, workload intensity may be a significant factor. Community pharmacists within this income bracket might work in environments with high patient volumes and heavy dispensing duties, resulting in time constraints that make reporting adverse drug reactions a lower priority. Previous research has shown that one of the main obstacles to healthcare professionals reporting ADRs is a heavy workload (Lopez-Gonzalez et al., 2009; N et al., 2025). However, more research is needed to determine the specific barriers faced by community pharmacists earning these wages when reporting adverse drug reactions.

The findings of this study indicate a significant difference in ADR reporting intentions between Saudi and non-Saudi community pharmacists, with non-Saudi pharmacists demonstrating a higher intention to report ADRs (72% vs 60%, p = 0.008). Several factors may explain this disparity, particularly differences in education, professional experience, and workplace culture. Non-Saudi pharmacists often receive their education in countries with well-established pharmacovigilance systems and curricula that emphasize ADR reporting as a core component of pharmaceutical care, such as the Philippines, India, or Egypt (Abdulsalim et al., 2023). This educational background may equip them with greater awareness, technical knowledge, and confidence to engage in reporting practices. In contrast, while pharmacy education in Saudi Arabia is evolving, it may still place less emphasis on pharmacovigilance training at the undergraduate level, potentially leading to lower familiarity with and intention to report ADRs. Additionally, non-Saudi pharmacists may have practical experience in healthcare systems where ADR reporting is embedded in professional norms and enforced by regulatory bodies. This cultural orientation toward accountability and adherence to professional standards may foster a stronger sense of duty to report ADRs, especially when practicing in a host country with rigorous healthcare expectations like Saudi Arabia. However, further research is needed to identify the underlying variables contributing to this gap in ADR reporting intentions between Saudi and non-Saudi pharmacists.

The TPB was effectively utilized in this study to analyze the factors influencing ADR reporting among community pharmacists. The TPB, with the addition of perceived moral obligation, explained approximately 58% of the variance in the intention of community pharmacists to report ADRs to the National Pharmacovigilance Center (NPC), underscoring the efficacy of this theoretical framework in understanding and predicting behavior in the context of pharmacovigilance. The study found that TPB constructs (attitude, subjective norms, and perceived behavioral control) as well as perceived moral obligation, were significantly associated with the intention of community pharmacists to report ADRs to the NPC in Saudi Arabia.

Community pharmacists who held a positive attitude toward reporting ADRs were more likely to intend to report them to the NPC. This finding aligns with results from studies conducted in the United States and Malaysia (Gavaza et al., 2011; Raghvan and Fatokun, 2021), highlighting the crucial role of perceptions in influencing pharmacists’ behavior regarding ADR reporting in Saudi Arabia. This suggests that fostering a positive attitude toward ADR reporting can potentially enhance community pharmacists’ willingness and commitment to report. Therefore, interventions aimed at promoting positive perceptions of ADR reporting among community pharmacists could be instrumental in improving pharmacovigilance practices and ultimately enhancing medication safety in Saudi Arabia.

Furthermore, the construct of subjective norms was a significant predictor of community pharmacists’ intention to report ADRs to the NPC in Saudi Arabia, which is consistent with previous studies (Godin et al., 2008; Gavaza et al., 2011; Raghvan and Fatokun, 2021). Subjective norms, reflecting an individual’s perception of societal expectations and pressures, evidently play a pivotal role in determining pharmacists’ intentions to engage in this important reporting process. These findings suggest that fostering a supportive social environment, where reporting ADRs is perceived as a normative and valued practice among peers and colleagues, can potentially enhance pharmacovigilance efforts within the community pharmacy setting. Therefore, strategies aimed at enhancing positive subjective norms, such as promoting a culture of reporting and providing support for reporting behaviors, may effectively increase pharmacists’ intentions to report ADRs.

This study indicated that the perceived behavioral control construct was a significant predictor of community pharmacists’ intention to report ADRs in Saudi Arabia. Community pharmacists who believed they had complete control and autonomy over reporting ADRs were more likely to report ADRs to the NPC. This finding contrasts with those of other studies conducted in different countries (Gavaza et al., 2011; Raghvan and Fatokun, 2021). It suggests that the influence of perceived behavioral control on the intention to report ADRs may vary across different cultural, regulatory, and healthcare system contexts. In Saudi Arabia, community pharmacists who feel empowered to make decisions regarding the reporting of ADRs demonstrate a strong intention to do so; however, this may not hold true in other countries, where factors such as regulatory frameworks, professional norms, or organizational structures may differ.

This study indicated that perceived moral obligation was a significant predictor of community pharmacists’ intention to report ADRs to the NPC in Saudi Arabia. Community pharmacists who perceived a high moral obligation were more likely to have a strong intention to report ADRs to the NPC, which aligns with prior research (Gavaza et al., 2011; Raghvan and Fatokun, 2021). This suggests that fostering a sense of moral duty among community pharmacists could enhance pharmacovigilance efforts and ultimately contribute to improving patient safety and healthcare quality in Saudi Arabia. Therefore, interventions aimed at strengthening moral awareness and responsibility within the pharmacy profession may help improve pharmacovigilance behavior among community pharmacists.

## 5 Limitations

Several limitations should be acknowledged. First, conclusions regarding causality are limited due to the use of a cross-sectional design in this study. Second, the use of a self-report survey may introduce recall bias, potentially overstating or understating the strength of the observed correlations. Third, the study focused exclusively on community pharmacists, suggesting the need for further research involving pharmacists from various practice settings. Fourth, the majority of participants were from the western region; the lower representation from other regions may limit the generalizability of the findings across Saudi Arabia. While the results provide valuable insights into the western region, caution is warranted when applying these findings to other parts of the country. To assess the impact of this overrepresentation, we conducted a stratified analysis by region (see Supplementary Table S5). Future studies should adopt stratified random sampling techniques or apply appropriate post-stratification weights to improve representativeness and minimize bias. Fifth, while this study reported that 27.4% of community pharmacists had previously submitted ADR reports to the NPC, it did not collect data on the number of reports ADRs submitted per individual pharmacist, which may limit the depth of understanding regarding actual reporting behavior. Sixth, Although the importance of timely ADR reporting was highlighted in the introduction, the study did not evaluate the time interval between the occurrence of ADRs and their submission to the NPC. These factors may influence reporting timelines and warrant further investigation. Additionally, while the instrument included items related to serious ADRs adapted from validated sources, the study did not assess pharmacists’ knowledge or understanding of the distinction between serious and non-serious ADRs, as the scope was limited to behavioral intentions. Future research is recommended to address these gaps and provide a more comprehensive understanding of ADR reporting practices among community pharmacists. Moreover, the lack of standardization of the TPB instrument used to assess pharmacists’ reporting behavior is another limitation. Lastly, the use of a convenience sampling method and online distribution of the survey prevented the calculation of a response rate. It was not possible to track the total number of pharmacists who received the invitation to participate, as the survey link was distributed through multiple channels, including social media and chain pharmacies. Future research that tracks survey invitations and completions or employs a more systematic sampling method would better enable evaluation of response rates and potential biases.

## 6 Conclusion

This study’s effective integration of the TPB and perceived moral obligation into its theoretical framework demonstrates the value of applying and adapting established models to comprehensively understand complex phenomena in healthcare research. By analyzing the factors influencing community pharmacists’ intentions to report ADRs in Saudi Arabia, the study underscores the pivotal role these healthcare professionals play in pharmacovigilance. Although the majority expressed a strong intention to report ADRs to the NPC, the alarmingly low actual reporting rate of 27% highlights the urgent need for a multifaceted strategy. This strategy should include targeted educational initiatives, awareness campaigns, continuous training, the cultivation of positive attitudes toward reporting, the promotion of supportive social norms within the pharmacy profession, and the reinforcement of a sense of moral obligation. The study’s findings carry important implications for policymakers and healthcare authorities in Saudi Arabia, offering insights to guide the development of strategies that enhance pharmacovigilance practices, improve medication safety, and ultimately strengthen patient care through more effective ADR reporting by community pharmacists.

## Data Availability

The raw data supporting the conclusions of this article will be made available by the authors, without undue reservation.

## Abbreviations

ADRs, Adverse Drug Reactions; NPC, National Pharmacovigilance Center; SFDA, Saudi Food and Drug Authority; HCPs, Healthcare Providers; WHO, World Health Organization; TPB, Theory of Planned Behavior.
