# Supplementary material for: Understanding community pharmacists’ intentions to report adverse drug reactions in Saudi Arabia: a theory of planned behavior analysis
Source: Front Pharmacol. 2025 May 20;16:1574412. doi: 10.3389/fphar.2025.1574412 (PMC12129803; doi:10.3389/fphar.2025.1574412)
Supplement: Supplementary file 1 [file Table1.docx]

**Supplementary Table 1.** Predictors associated with community pharmacists’ intention to report ADRs to NPC stratified analysis by region

| **Region of work in Saudi Arabia** | **TPB constructs and Perceived Moral Obligation** | **Odds Ratio** | **Lower 95% CI** | **Upper 95% CI** | **p-value** |
| --- | --- | --- | --- | --- | --- |
|  |  |  |  |  |  |
| Western region | Attitude | 1.149 | 1.077 | 1.226 | < 0.001 |
|  | Subjective Norms | 1.228 | 1.098 | 1.374 | < 0.001 |
|  | Perceived Behavioral Control | 1.107 | 0.947 | 1.295 | 0.202 |
|  | Perceived Moral Obligation | 1.465 | 0.954 | 2.250 | 0.081 |
| Eastern region | Attitude | 1.055 | 0.931 | 1.195 | 0.405 |
|  | Subjective Norms | 1.438 | 0.926 | 2.231 | 0.106 |
|  | Perceived Behavioral Control | 1.023 | 0.652 | 1.606 | 0.920 |
|  | Perceived Moral Obligation | 1.809 | 0.489 | 6.692 | 0.375 |
| Central region | Attitude | 1.184 | 1.059 | 1.325 | 0.003 |
|  | Subjective Norms | 1.734 | 1.298 | 2.317 | < 0.001 |
|  | Perceived Behavioral Control | 1.413 | 1.021 | 1.957 | 0.037 |
|  | Perceived Moral Obligation | 1.091 | 0.589 | 2.019 | 0.782 |
| Northern region | Attitude | 1.729 | 0.686 | 4.355 | 0.246 |
|  | Subjective Norms | 1.792 | 0.767 | 4.188 | 0.178 |
|  | Perceived Behavioral Control | 6.307 | 0.309 | 128.558 | 0.231 |
|  | Perceived Moral Obligation | 252.110 | 0.039 | 1626261.33 | 0.217 |
| Southern region | Attitude | 1.166 | 1.074 | 1.267 | < 0.001 |
|  | Subjective Norms | 1.132 | 0.941 | 1.362 | 0.189 |
|  | Perceived Behavioral Control | 1.228 | 0.926 | 1.627 | 0.153 |
|  | Perceived Moral Obligation | 1.244 | 0.748 | 2.071 | 0.400 |
