# Supplementary material for: Understanding community pharmacists’ intentions to report adverse drug reactions in Saudi Arabia: a theory of planned behavior analysis
Source: Front Pharmacol. 2025 May 20;16:1574412. doi: 10.3389/fphar.2025.1574412 (PMC12129803; doi:10.3389/fphar.2025.1574412)
Supplement: Supplementary file 2 [file DataSheet1.pdf]

Factors Affecting Community Pharmacists in Saudi Arabia to Report Adverse Drug Reactions (ADRs) using the Theory of Planned Behavior

Dear Pharmacist,

We are conducting a scientific study aimed at evaluating community pharmacists' intentions and perspectives regarding the reporting of adverse drug reactions (ADRs) to the Saudi Food and Drug Authority's National Pharmacovigilance Center (NPC).

We kindly request your participation by completing a short, structured questionnaire.

Your input is valuable and will contribute significantly to the understanding and enhancement of pharmacovigilance practices in Saudi Arabia.

The questionnaire will take approximately 10 minutes to complete.

Participation is entirely voluntary, and you may withdraw from the study at any time without any penalty or consequence.

Please be assured that all responses will remain anonymous, and the collected data will be treated with strict confidentiality and used solely for academic and research purposes.

This study has received ethical approval from the Research Ethics Committee at Taif University (Approval Number: 45-046).

If you have any questions or require further information regarding this research, please feel free to contact us at:

Email: F.Alsulami@tu.edu.sa

1. Do you agree to participate in this research?

	Yes
	No

2. Do you hold a valid pharmacist registration license issued by the Saudi Commission for Health Specialties (SCFHS)?

	Yes
	No

3. Please indicate your gender.

	Male
	Female

4. Please indicate your age.

…………………

5. Please indicate your nationality.

	Saudi
	Non-Saudi

6. Please indicate your highest educational degree that you have attained.

	Bachelor's Degree in Pharmacy
	Doctor of Pharmacy (PharmD)
	Higher Education Degree in Pharmacy (MS or PhD)

7. Please indicate your region of work in Saudi Arabia

	Western region
	Eastern region
	Central region
	Northern region
	Southern region

8. Please indicate your monthly income.

	6,999 SAR or less.
	7,000 SAR to 10,999 SAR
	11,000 SAR to 14,999 SAR
	15,000 SAR or More.

9. Please specify the number of years since you were registered as a pharmacist in Saudi Arabia.

	Less than 5 years
	From 5 years to 10 years
	More than 10 years

10. Are you aware of the existence of National Pharmacovigilance Centre (NPC) in Saudi Arabia?

	Yes
	No

11. Are you familiar with the way of adverse drug reactions (ADRs) reporting in Saudi Arabia?

	Yes
	No

12. Have you seen the adverse drug reactions (ADRs) reporting form before?

	Yes
	No

13. Have you been trained to report adverse drug reactions (ADRs) before?

	Yes
	No

Theory of planned behavior

Section A: Intention to report ADRs *(Please select one box only)*

Intentions	1<br>Extremely Unlikely	2<br>Moderately Unlikely	3<br>Unlikely	4<br>Neutral	5<br>Likely	6<br>Moderately Likely	7<br>Extremely Likely
I intend to report serious ADRs that I will encounter to the Saudi NPC.							
I will try to report serious ADRs that I will encounter to the Saudi NPC.							
I plan to report serious ADRs that I will encounter to the Saudi NPC.							

Section B: Beliefs about ADRs reporting *(Please select one box only)*

Attitude

**Attitude**	**-3**<br>**Extremely Unlikely**	**-2**<br>**Moderately Unlikely**	**-1**<br>**Unlikely**	**0**<br>**Neutral**	**1**<br>**Likely**	**2**<br>**Moderately Likely**	**3**<br>**Extremely Likely**
Reporting ADRs to NPC in Saudi Arabia is valuable.							
Reporting ADRs to NPC in Saudi Arabia is pleasant.							
Reporting ADRs to NPC in Saudi Arabia is good.							
Reporting ADRs to NPC in Saudi Arabia is enjoyable.							
Reporting ADRs to NPC in Saudi Arabia is beneficial.							

Subjective Norms

**Subjective Norms**	**-3**	**-2**	**-1**	**0**	**1**	**2**	**3**
	**Extremely Unlikely**	**Moderately Unlikely**	**Unlikely**	**Neutral**	**Likely**	**Moderately Likely**	**Extremely Likely**
Most people who are important to me think that I should report ADRs that I encounter to NPC in Saudi Arabia.							
The people in my life whose opinions I value would approve my reporting of ADRs that I encounter to NPC in Saudi Arabia.							
The pharmacists whose opinions I value report ADRs to NPC in Saudi Arabia.							

Perceived Behavioral Control

**Perceived Behavioral Control**	**-3**	**-2**	**-1**	**0**	**1**	**2**	**3**
	**Extremely Unlikely**	**Moderately Unlikely**	**Unlikely**	**Neutral**	**Likely**	**Moderately Likely**	**Extremely Likely**
You believe you have complete control over reporting ADRs that you encounter to NPC in Saudi Arabia.							
It is mostly up to me whether or not I report ADRs to NPC in Saudi Arabia.							

Section D: Perceived moral obligation ***(Please select one box only)***

**Perceived moral obligation**	**1**	**2**	**3**	**4**	**5**	**6**	**7**
	**Extremely Unlikely**	**Moderately Unlikely**	**Unlikely**	**Neutral**	**Likely**	**Moderately Likely**	**Extremely Likely**
I believe I have a moral obligation to report ADRs that I will encounter to NPC in Saudi Arabia.							
